# IL-1β promotes IL-9-producing Th cell differentiation in IL-2-limiting conditions through the inhibition of BCL6

**DOI:** 10.3389/fimmu.2022.1032618

**Published:** 2022-11-01

**Authors:** D. Alejandro Canaria, Maia G. Clare, Bingyu Yan, Charlotte B. Campbell, Zachariah A. Ismaio, Nicole L. Anderson, Sungtae Park, Alexander L. Dent, Majid Kazemian, Matthew R. Olson

**Affiliations:** ^1^ Department of Biological Sciences, Purdue University, West Lafayette, IN, United States; ^2^ Department of Biochemistry, Purdue University, West Lafayette, IN, United States; ^3^ Department of Microbiology and Immunology, Indiana University School of Medicine, Indianapolis, IN, United States; ^4^ Department of Computer Science, Purdue University, West Lafayette, IN, United States

**Keywords:** Th cell differentiation, IL-9 cytokine, IL-2 signaling, nuclear factor-kB, Th9 cells

## Abstract

IL-9-producing CD4^+^ T helper cells, termed Th9 cells, differentiate from naïve precursor cells in response to a combination of cytokine and cell surface receptor signals that are elevated in inflamed tissues. After differentiation, Th9 cells accumulate in these tissues where they exacerbate allergic and intestinal disease or enhance anti-parasite and anti-tumor immunity. Previous work indicates that the differentiation of Th9 cells requires the inflammatory cytokines IL-4 and TGF-β and is also dependent of the T cell growth factor IL-2. While the roles of IL-4 and TGF-β-mediated signaling are relatively well understood, how IL-2 signaling contributes to Th9 cell differentiation outside of directly inducing the *Il9* locus remains less clear. We show here that murine Th9 cells that differentiate in IL-2-limiting conditions exhibit reduced IL-9 production, diminished NF-kB activation and a reduced NF-kB-associated transcriptional signature, suggesting that IL-2 signaling is required for optimal NF-kB activation in Th9 cells. Interestingly, both IL-9 production and the NF-kB transcriptional signature could be rescued by addition of the NF-kB-activating cytokine IL-1β to IL-2-limiting cultures. IL-1β was unique among NF-kB-activating factors in its ability to rescue Th9 differentiation as IL-2 deprived Th9 cells selectively induced IL-1R expression and IL-1β/IL-1R1 signaling enhanced the sensitivity of Th9 cells to limiting amounts of IL-2 by suppressing expression of the Th9 inhibitory factor BCL6. These data shed new light on the intertwined nature of IL-2 and NF-kB signaling pathways in differentiating Th cells and elucidate the potential mechanisms that promote Th9 inflammatory function in IL-2-limiting conditions.

## Introduction

CD4^+^ T helper (Th) cells differentiate from a pool of naïve quiescent precursors into cytokine-producing effector cells in response to a complex set of cell surface receptor and cytokine cues. For IL-9-secreting Th cells, termed Th9 cells, this involves induction of TNF receptor family member co-stimulatory signals (i.e. CD28, OX40, GITR) along with specific cytokines including IL-4, TGF-β (1–4). The combination of these signals induces a downstream transcription factor network centered around STAT6, IRF4, BATF, and PU.1 that endows these cells with a unique proliferative capacity and the production of high levels of IL-9 (2). Th9 cells provide optimal control of parasite infection and potent anti-cancer immunity after adoptive cell therapy (5–8). However, when dysregulated, these cells can also contribute to autoimmunity in the form of allergic airway disease and ulcerative colitis (9, 10). Therefore, understanding the factors and cellular pathways that drive Th9 cell differentiation is critical to develop novel therapies that enhance anti-tumor immunity or thwart autoimmunity.

IL-2 is rapidly produced by T cells after T cell receptor activation/CD28 co-stimulation and is a potent activator of STAT5 and T cell proliferation (11, 12). Beyond its role in proliferation, STAT5 promotes the differentiation of Th9 cells by directly binding and transactivating the *Il9* locus and allowing for epigenetic remodeling of upstream regulatory regions (13). IL-2/STAT5 signaling also contributes to Th9 cell differentiation through transcriptional repression where STAT5 represses expression of BCL6 that competes for STAT5 binding at the *Il9* locus and limits Th9 differentiation (14, 15). Further, we demonstrated that STAT5 suppresses a Th17-like phenotype in differentiating Th9 cells (16). Despite the importance of IL-2 in the differentiation process, we and others have noted that IL-9-producing Th cells often develop and act to induce protective or auto-immunity in IL-2-limiting environments. For example, Th9 cells maintain potent inflammatory activity despite IL-2 sequestration by tumor- or tissue-resident T regulatory cells (17–19). Similarly, Th9 cells co-develop with IL-2/STAT5-inhibited Th17 cells in settings of chronic fungal- or house dust mite-driven allergic disease and maintain a strong Th9-associated gene signature in HDM allergic patients (20–22). Together, these data suggest that additional signals may be involved in promoting Th9 cell differentiation when IL-2 is limiting.

Heightened NF-kB activity is another hallmark of activated T cells and is thought to precede IL-2 production upon initial T cell priming. During the process of T cell activation, both canonical (p50/c-rel/p65) and non-canonical (p52/Relb) NF-kB-activating pathways are induced after ligation of co-stimulatory receptors (i.e. CD28) (23–27). Co-receptor ligation leads to the nuclear localization of NF-kB subunits where they pair with NFAT to drive the production of IL-2 and several other early activation-associated genes (27). During T helper cell differentiation, NF-kB generally promotes the differentiation of all Th cell lineages by cooperating with lineage-associated STAT factors and “master” TFs to activate gene transcription (27). In Th1, Th2 and Th17 differentiation programs, the canonical NF-kB pathway plays a predominant role (28–30) whereas Th9 cells exhibit activation of both canonical and non-canonical pathways (3). NF-kB activation is prolonged in Th9 cells as compared to other Th lineages and suggests a unique role for this pathway in Th9 differentiation (31). Indeed, enhancing the activation of NF-kB *via* cytokines (TNF, TL1A) or TNFR family members (OX40, GITR, DR3) augments Th9 cell differentiation and enhances Th9-mediated inflammatory potential (3, 32–34). Of note, many NF-kB-activating factors are often elevated in tumors and inflamed tissues where IL-2 availability is thought to be limiting (3, 35–38) and suggests that engagement of the NF-kB pathway may act to induce or maintain the Th9 phenotype in these IL-2-limited environments.

While both IL-2 signaling and NF-kB are induced by co-stimulatory signals and required for optimal Th9 cell differentiation, the interplay between IL-2 and NF-kB signaling in this process is not well understood. In this report, we established a critical link between IL-2/STAT5 signaling and the induction of the NF-kB transcriptional signature in differentiating Th9 cells. When Th9 cells were cultured in the absence of IL-2 or under IL-2-limiting conditions, we observed a coordinate loss of both IL-9 production and an NF-kB-targeted transcriptome, both of which could be rescued by addition of the exogenous NF-kB-activating cytokine IL-1β. Interestingly, IL-1β was unique among the NF-kB-activating factors we tested for its ability to rescue IL-9 production due to the specific upregulation of the IL-1 receptor (IL-1R1) in the absence of IL-2. IL-1β additionally repressed the expression of the Th9 inhibitory transcription factor BCL6 and ectopic BCL6 expression greatly diminished the IL-1β-mediated rescue of IL-9. These data implicate a feedback loop where IL-2-deprived Th9 cells upregulate IL-1R1 and enhance their capacity to respond to IL-1β. In turn, IL-1β-mediated activation of NF-kB limits the expression of the anti-Th9 factor BCL6 which results in enhanced IL-2 responsiveness and IL-9 production. This model addresses the seeming paradox in which IL-2 is required for *in vitro* Th9 differentiation, but Th9 cells are often found in inflamed tissues *in vivo* where IL-2 is limited.

## Materials and methods

### Mice

C57BL/6 and OT-II transgenic mice were originally acquired from Jackson laboratories and bred and housed in our AAALAC-accredited Purdue University animal housing facilities under a 12 hour on, 12 hour off light cycle. All mice received autoclaved T.2018SC.15 18% protein chow (Envigo) and autoclaved drinking water ad libitum. The animal protocol was established according to Purdue University Institutional Animal Care and Use Committee (IACUC) guidelines. The experiments done here were done in compliance with a Purdue Animal Care and Use Committee-approved protocol.

### Naïve CD4 T cell isolation and cell culture

Spleens and lymph nodes from C57BL/6 and OT-II mice were mechanically dissociated with frosted slides to generate a single cell suspension for CD44-negative naïve CD4 T cell isolation, using the Naïve CD4+ T cell Isolation Kit (Miltenyi Biotec, Auburn, CA) according to manufacturer’s protocol. Naïve CD4 T cell (CD4^+^, CD44^low^, CD62L^hi^) purity was >90% as measured by an Attune NxT Flow cytometer (Thermo Fisher Scientific). After isolation, a total of 1x10^6^ cells/mL were cultured in a 1:1 combination of RPMI and DMEM supplemented with 1% penicillin/streptomycin (Life Technologies), 10% FBS, and 50 mM 2-ME (Life Technologies). The cells were cultured at 37°C for 4 days in plates coated with anti-CD3-(2μg/mL, 2C11, Bio X cell), with soluble aCD28 (4μg/mL, 37.51, Bio X cell), and the Th9-polarizing conditions: anti-IFNγ(10μg/mL), IL-4(20ng/mL), TGFβ(4ng/mL), anti-IL-10R(10μg/mL). IL-2 was neutralized using anti-IL-2 and anti-CD25 antibodies as previously published (16). In some conditions, we supplemented the cultures with NF-kB activators IL-1β (20ng/mL), IL-33 (20ng/mL), PAM3CSK4 (1μg/mL), DTA1 (5μg/mL) and OX40L (5μg/mL), and we used the NF-kB inhibitors QNZ (2nM, Cayman Chemical) and BAY 11-7082 (0.5μM, Cayman Chemical) with the respective dilution of DMSO as vehicle control. After 4 days in culture, we assessed cell number and cell viability in each well using trypan blue cell counting on a hemocytometer and the cytokine and TF expression through flow cytometry as previously described (16).

### Intracellular cytokine and transcription factor staining

After culture, 1X10^6^ cells/mL in each condition were used for intracellular cytokine staining (ICS) and/or transcription factor (TF) staining. For ICS, harvested differentiated Th9 cells were stimulated with PMA (0.5μg/mL) and ionomycin (0.5ug/ml, Sigma-Aldrich) for 2.5h at 37°C using U-bottom 96-well plates. After 2.5 hours, monensin (2μM final concentration, BioLegend) was added to each well for an additional 2.5-hour incubation. After that, the cells were collected and stained with fixable viability dye (eFluor780, Thermo Fisher Scientific) and mouse anti-CD4 antibody (RM4-5, 1μg/mL, Thermo Fisher Scientific), for retroviral transductions experiments the cells were also stained with anti-H2-K^k^ antibody (36-7-5, 5μg/mL, BioLegend). The cells were subsequently fixed with 3% formaldehyde for 15 min. After fixation, the cells were fixed again with True-Nuclear Fixation Buffer (BioLegend) for 45 min at room temperature and subsequently permeabilized with True-Nuclear Permeabilization Buffer (BioLegend) as per manufacturer’s instructions. The cells were then stained with antibodies targeting the cytokines: IL-9 (RM9A4, 1μg/mL, BioLegend) IL-17A (TC11-18H10.1, 1μg/mL, BioLegend) IL-2 (JES6-5II4, 1μg/mL BioLegend) and transcription factors BCL6 (K112-91, 1μL/test, BD Pharmingen), and IRF4 (IRF4.3E4, 0.5μg/ml, BioLegend) for 30 min, washed, and resuspended in FACS buffer for performing flow cytometry analyses.

### Phospho-p65 staining

Naïve CD4 T cells were cultured as above using Th9 polarizing conditions with or without anti-IL-2/CD25. After culture, cells were collected and stained with anti-CD4 antibody (RM4-5, 1μg/mL, Thermo Fisher Scientific) and fixable viability dye (eFluor780, Thermo Fisher Scientific) for 15 minutes in 1xPBS at 4°C. Cells were washed with 200μL of PBS and fixed with 1.5% formaldehyde at room temperature for 10 minutes. Cells were washed with 1xPBS and treated with 100% ice-cold methanol for 1 hour at 4°C. Subsequently, the cells were pelleted at 1200xg for 10 min at 4°C, washed with 1xPBS and stained in FACS buffer for 1h at 4°C with Phospho-NF-kB p65(ser529) antibody (NfkBp65S529-H3, Invitrogen ref MA5-37414) as per manufacturer’s recommended amount per test (5μl/1X10^6^ cells). Cells where then washed, resuspended in FACS buffer for flow cytometric analyses.

### RNA isolation and qRT-PCR

After *in vitro* polarization in the conditions listed above, we isolated RNA using TRIzol™ Reagent (Thermo Fisher) as per manufacturer’s instructions. After quantifying RNA concentration using a NanoDrop™ ONE-W (Thermo Fisher Scientific) spectrophotometer, 1000ng of RNA were reverse transcribed using the High Capacity cDNA Reverse Transcription Kit (Thermo Fisher Scientific) and used for quantitative PCR analysis using theTaqMAN™ Fast Advanced Master Mix (Thermo Fisher Scientific) and the probes: Il9(Mm00433405_m1), Batf3(mM01318274_m1), Sgk1(Mm00441380_m1), Atf3(Mm00476033_m1), Csf1(Mm00432686_m1), Bcl6(Mm00477633_m1), Il1r1(Mm00434237_m1), Il1rap(Mm00492638_m1), Il1rl1(Mm00516117_m1), Tlr2(Mm01213946_g1), Tnfrsf4,(Mm00442039_m1), Tnfrsf18(Mm00437136_m1), (all from Thermo Fisher Scientific), and a Bio-rad CFX96 system as per manufacturer’s instructions. Relative expression analysis was performed using the 2^-ΔΔct^ method and data were quantified and plotted using GraphPad Prism V9.4.0 software.

### Ovalbumin-induced allergy *in vivo* model

Naïve CD4 T cells where isolated from OT-II TCR-transgenic mice as per above, and the resulting cells were cultured under Th9 conditions, with or without the addition of IL-1β and with or without IL-2 neutralization as mentioned above. At 4 days after culture, the cells were rested in media for 24 hours, followed by subsequent washing (2 washes with 10 mL sterile PBS), counted *via* hemocytometer and 0.5 X10^6^ cells were retro-orbitally injected into lightly isoflurane-anesthetized C57BL/6 mice. At 24 hours post-adoptive transfer, the mice were treated intranasally with recombinant chicken ovalbumin protein (100 μg in 25μl of 1xPBS, Sigma Aldrich) and TSLP (200 ng in 25 μl of 1xPBS, Sino Biological) daily for 5 consecutive days. Mice treated with IL-9 neutralizing antibody (9C1, BioXcell) were treated daily with 100μg in 50μL of PBS before the OVA/TSLP challenge. Twenty-four hours after the last intranasal treatment, the mice were euthanized using cervical dislocation and dissected for lung tissue collection.

### Lung digestion, cell isolation and stimulation

Lungs from OVA-induced allergy mice were perfused with 1X PBS and dissected. The lungs were placed in FACS tubes with digestion buffer (1.5mg/ml type III collagenase (Worthington), 100ng/mL DNase I, RPMI media and 10% Newborn Calf serum) and sliced in 2mm^2^ tissue pieces using dissecting scissors. Minced lung tissue was then digested for 50 minutes in a rotor incubator at 37°C. After digestion, the digested tissue was transferred to a 70μm nylon cell strainer (USA Scientific) to remove debris. The cell suspension was then treated with 155mM Ammonium Chloride, 12mN Sodium Bicarbonate, 0.1mM EDTA buffer to lyse red blood cells and mononuclear cells were enriched from this suspension *via* centrifugation (500xg for 10 min) through 40% Percoll (GE Healthcare). Pelleted cells were resuspended in complete RPMI media and counted before staining for the inflammatory cell markers: CD45(30-F11, 0.2μg/mL, BioLegend), CD3(17A2, 1μg/mL, BioLegend), CD19 (6D5, 1μg/mL, BioLegend), NK1.1(PK136 1μg/mL, BioLegend), Siglec-F (1RNM44N, 1 μg/mL, Invitrogen), Ly6G(RB6-8C5, 1μg/mL, BioLegend), CD11c(N418, 1 μg/mL, BioLegend), CD11b (M1/70, 1 μg/mL, BioLegend) for assessing immune cells infiltrating the lungs using flow cytometry. For stimulated panel: 1X10^6^ cells were stimulated using PMA (0.5ng/mL) and ionomycin (0.5ug/ml, Sigma-Aldrich) for 4h at 37°C using U-bottom 96-well plates. After the 4h, monensin (2μM final concentration, BioLegend) was added to each well for an additional 2-hour incubation. After that, the cells were stained as per above using the surface markers: anti-CD90.2(53-2.1, 1.5μg/mL, BioLegend), anti-CD4(RM4-5, 1μg/mL, Thermo Fisher Scientific), anti-CD45(30-F11, 0.2μg/mL, BioLegend), anti-mouse-TCR Vα2 (B20.1, 1μg/mL, BioLegend), anti-mouse-TCR Vβ5 (MR9-4, 2μg/mL, BioLegend), fixable viability dye (eFluor780, Thermo Fisher Scientific) and intracellular anti-IL-9 (RM9A4, 1μg/mL, BioLegend). Stained cells were analyzed using an Attune NxT Flow cytometer (Thermo Fisher Scientific).

### Histology

Lung tissue was collected on day 6 after adoptive cell transfer and OVA treatment and stored in buffered formalin (Formal-Fixx, Thermo Fisher Scientific) as per manufacturer’s instructions. Then the samples were transferred to 70% ethanol, and submitted to HistoWiz (https://home.histowiz.com) for tissue sectioning, Hematoxylin and Eosin (H&E) staining and imaging.

### Retroviral transduction

The vector MSCV-H2-K^k^ (gifted by Mark Kaplan, Indiana School of Medicine) was utilized for generating viral particles for ectopic expression BCL6 as we have previously described (16). Briefly, Plat-E cells were transfected with 10μg of plasmid and their empty vector controls, and with 5μg of the packaging plasmid pCL-Eco (gifted by Dr. Mark Kaplan, Indiana School of Medicine) using Lipofectamine 2000 (Thermo Fisher Scientific) per the supplier’s instruction. At 48 hours post-transfection, the supernatant containing viral particles was harvested and cleared of cellular debris by centrifugation at 300xg for 10 minutes. Viral particle-containing supernatants were used for spin infection of 24 hour-activated Th9 cells in the presence of 8μg/ml of polybrene at 500xg for 1.5h at 37°C. Infected cells were harvested at 72 hours post-viral transduction for intracellular staining and flow cytometry as above.

### RNA-seq analysis

The raw files of sequencing read are downloaded from GSE41317. Gene expression levels in each library were quantified by “rsem-calculate-expression” by RSEM v1.3.0 (39) with default parameters except “—bowtie-n 1 –bowtie-m 100 –seed-length 28”. The bowtie index (GRCm38) required by RSEM software was prepared by “rsem-prepare-reference” on all RefSeq genes, downloaded from UCSC table browser in April 2017. EdgeR v3.24.3 (40) package was utilized to identify differentially expressed genes (DEGs) between IL-2 knock-out and wildtype samples. Gene set enrichment analysis (GSEA) was performed using GSEA “investigate Gene Sets” (41) with top 500 differentially expressed genes (DEGs) in all hallmark gene sets.

## Results

### IL-2/STAT5 signaling is required for optimal NF-kB signaling in Th9 cells

**Figure 1:**
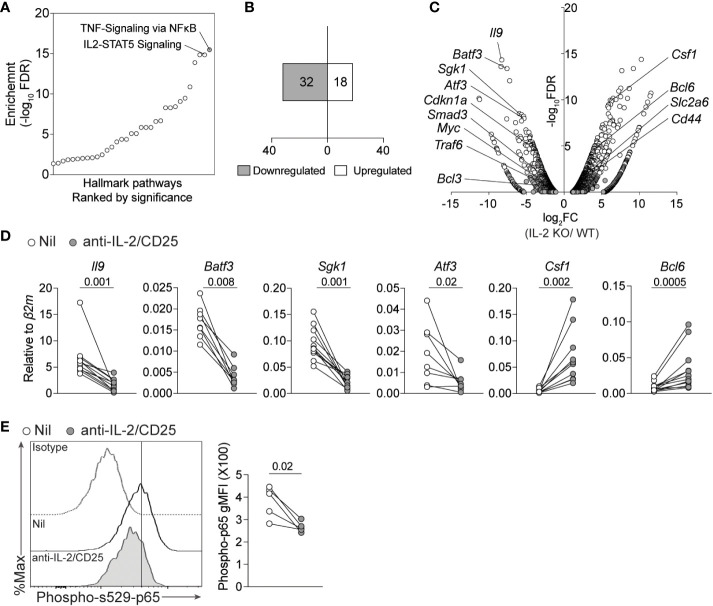
IL-2 deficiency results in a reduced NF-kB-associated gene signature in Th9 cells. **(A)** Pathway analysis from bulk-RNA-seq analysis of Th9 cells polarized from WT or IL-2-KO mice previously published (*GSE41317).* **(B)** Number of up- and down- regulated NF-kB signature genes (Fold change >2, p-value<0.05). **(C)** Volcano plot depicting selected genes regulated by NF-kB that are differentially expressed in IL-2 KO vs WT Th9 cells. **(D)** Th9 cells were cultured in standard or IL-2-limiting conditions, and 4 days after initial activation total RNA was isolated for qPCR validation of selected NF-kB-associated genes obtained from panel **(A)** Data in panel **(D)** are representative of naive T cell cultures from 8-11 individual mice pooled from 3 individual experiments. **(E)**. Representative histograms and quantified geometric Mean Fluorescence Intensity (gMFI) of phospho- s529-p65 staining in cells cultured as in **(D)** Each data point represents values obtained from T cells isolated from one mouse, for a total of n=5 mice. Data were considered significantly different when paired *t*-test *p* value was ≤0.05.

We, and others, have previously demonstrated that IL-2 signaling and downstream STAT5 activation was required for the differentiation of Th9 cells and IL-9 production (15, 16, 42, 43). While STAT5 directly binds a number of conserved regions upstream of the *Il9* locus, STAT5 also indirectly promotes the Th9 phenotype by suppressing a Th17-like phenotype and limiting expression of the repressive factor BCL6 (14, 15). However, these indirect mechanisms are incompletely understood. To address this, we initially assessed cellular pathways that were altered in WT and IL-2-deficient Th9 cells (15). Surprisingly, the top pathway that was altered in IL-2-deficient Th9 cells was related to NF-kB signaling, followed by IL-2/STAT5 signaling (**Figure 1A**). These data are meaningful as recent studies identified an important role for NF-kB in promoting IL-9 expression in various conditions and enhancing their inflammatory activity *in vivo* (3, 32–34). Consistent with this role in promoting the Th9 phenotype, the majority (64%) of genes within the 50 identified NF-kB signature exhibited reduced expression as compared to WT cells (i.e. *Sgk1, Atf3, Batf3*) whereas relatively fewer NF-kB-related genes were enhanced (i.e. *Csf1*) in the absence of IL-2 (**Figures 1B, C**). We validated these data *via* qPCR in our laboratory with Th9 cells that were cultured normally or under IL-2-limiting conditions (i.e. IL-2 and CD25 blockade) and observed significantly decreased expression of *Il9*, *Sgk1*, *Atf3*, and *Batf3* when IL-2 was limiting (**Figure 1D**). In agreement with our RNA-seq analysis, we also observed significantly increased expression of *Csf1* and *Bcl6* after IL-2 blockade (**Figures 1C, D**). Canonical NF-kB transcriptional regulation is mediated by the p50/p65 complex (RelA) which, under inactive conditions, is sequestered in the cytoplasm by IkBa. Activation of NF-kB requires degradation of IkBα and translocation of p65/p50 to the nucleus, both processes require phosphorylation (44, 45). To test whether the activity of NF-kB was altered in IL-2-sufficient or IL-2-depleted Th9 cells, we detected phosphorylated p65 in the residue Serine 529 using flow cytometry, which has been associated with increased transcriptional activity of NF-kB (46–48).We observed that phospho-S529-p65 was significantly reduced in IL-2-deprived Th9 cells after 4 days of culture (**Figure 1E**) indicating that IL-2 signaling is required for the induction or maintenance of active NF-kB in differentiating Th9 cells. Together, these data indicate that IL-2 signaling is critical for the activity of NF-kB and the induction of its target genes in Th9 cells.

### IL-1β signaling rescues IL-9 production in IL-2 limiting conditions *via* activation of NF-kB

**Figure 2:**
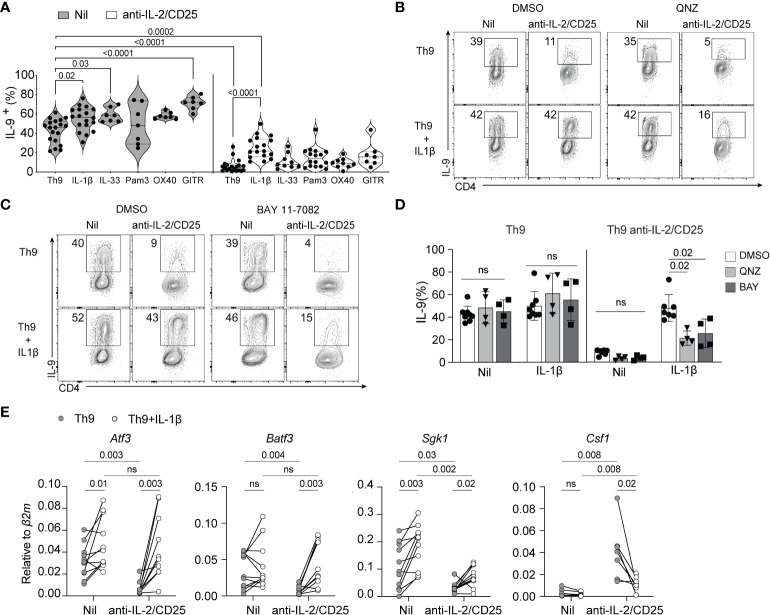
IL-1β-induced NF-kB rescues Th9 differentiation in IL-2-limiting conditions. **(A)** Th9 cells were cultured in standard or IL-2-limiting conditions in the presence of the indicated NF-kB-activating cell surface receptors and cytokines, and intracellular IL-9 was measured at day 5 of culture after restimulation of cells with PMA and ionomycin. Each data point represents cells from at least n=7 mice or more per group. **(B, C)** Representative contour plots of IL-9 production of Th9 and IL-2-deprived Th9 cells in the presence or absence of IL-1β and the NF-kB inhibitors QNZ (1nm) or BAY 11-7082 (2.5μM), depicting the NF-kB-dependency of IL-1β-mediated rescue of Th9 cell differentiation. **(D)** Quantification of IL-9^+^ Th cells in culture with NF-kB inhibitors, each data point represents naïve T cell cultures from individual mice n= 4 mice from drug-treated cells and n=7 for DMSO controls. **(E)** NF-kB-associated gene expression at day 5 of culture from non-restimulated Th9 and IL-2-deprived Th9 cells cultured in the presence or absence of IL-1β. Each point represents data from T cells isolated from at least 8 or more mice per group. Data are considered significant if *p* value ≤0.05 in a one-way ANOVA analysis with a Tukey post-test. Error bars represent standard deviation. ns, non-significant.

As IL-2 signaling was required for optimal induction of IL-9 and the NF-kB transcriptional signature, we hypothesized that restoration of NF-kB activity would rescue IL-9 production by these cells under IL-2 limiting conditions. To test this, we cultured naïve CD4 T cells under standard Th9 conditions with and without IL-2 blockade and further added NF-kB-activating cytokines (IL-1β, IL-33) or triggered the signaling of NF-kB-inducing receptors (TLR2 *via* PAM3CSK4, OX40 or GITR *via* crosslinking antibodies) that have been previously shown to enhance Th9 cell differentiation (3, 34, 49). Consistent with these previous reports, addition of IL-1β, IL-33 and crosslinking antibodies enhanced Th9 cell differentiation under IL-2 replete conditions. However, treatment with OX40 crosslinking antibodies only modestly enhanced IL-9 production and was not statistically significant (**Figure 2A**). Under IL-2-limiting conditions, only the addition of 10ng/ml of IL-1β significantly and consistently enhanced IL-9 production as compared to the untreated (e.g. Nil) control while all others NF-kB activators exhibited a limited capacity to rescue IL-9 production in these initial screening experiments (**Figure 2A**). For this reason, we have focused on IL-1β signaling for the remainder of our studies.

IL-1β binds its receptor, IL-1R1, and signals through MyD88 and TRAF6 which leads to the downstream activation of NF-kB and the MAPK pathway (50–55). As our RNA-seq analysis indicated that the NF-kB-associated transcriptional signature is affected by limited IL-2, we asked if NF-kB signaling was required for IL-1β-mediated rescue of IL-9 production. To this end, we cultured cells under standard or IL-2-limiting conditions as above in the presence or absence of an increased dose of IL-1β (20ng/ml, to enhance IL-9 rescue potential) and the NF-kB inhibitors QNZ or BAY110782 and assessed IL-9 production at the end of culture. Th9 cells cultured under IL-2 limiting conditions exhibited a similar reduction of IL-9 production and this was significantly rescued with addition of IL-1β in our vehicle control (DMSO)-treated cells now to standard Th9 levels, likely due to the increased amount of IL-1β used. Cells under IL-2 limiting conditions treated with IL-1β and QNZ or BAY117082 exhibited a 50% loss of IL-9 production, indicating that NF-kB activity was at least partially required for the rescuing effect of IL-1β (**Figures 2B-D**). Of note, the concentrations of drugs used in these studies was below that which has been published in other studies as we found higher inhibitor concentrations caused enhanced cell death under IL-2-limiting conditions. Therefore, the degree of NF-kB dependency we have measured here is likely underestimated.

### IL-1β normalizes the expression of selected NF-kB genes in IL-2 limiting conditions

We showed above that IL-1β rescues IL-9 production in IL-2-limiting conditions, but how this impacts other NF-kB-regulated genes in the signature we identified remains unclear. To address this, we performed qPCR of select NF-kB-associated genes (*Batf3, Atf3, Sgk1*, and *Csf1*) that were differentially regulated in IL-2-deficient Th9 cells as depicted above (**Figures 1C, D**). IL-1β was able to normalize the expression of genes such as*, Batf3, Atf3* and *Csf1* (**Figure 2D**). However, the expression of *Sgk1*, was only marginally increased as compared to controls (**Figure 2D**). These data indicate that IL-1β, at least in part, normalizes the IL-2-dependent NF-kB gene signature in Th9 cells.

### IL-1β rescues the *in vivo* inflammatory activity of Th9 cells

**Figure 3:**
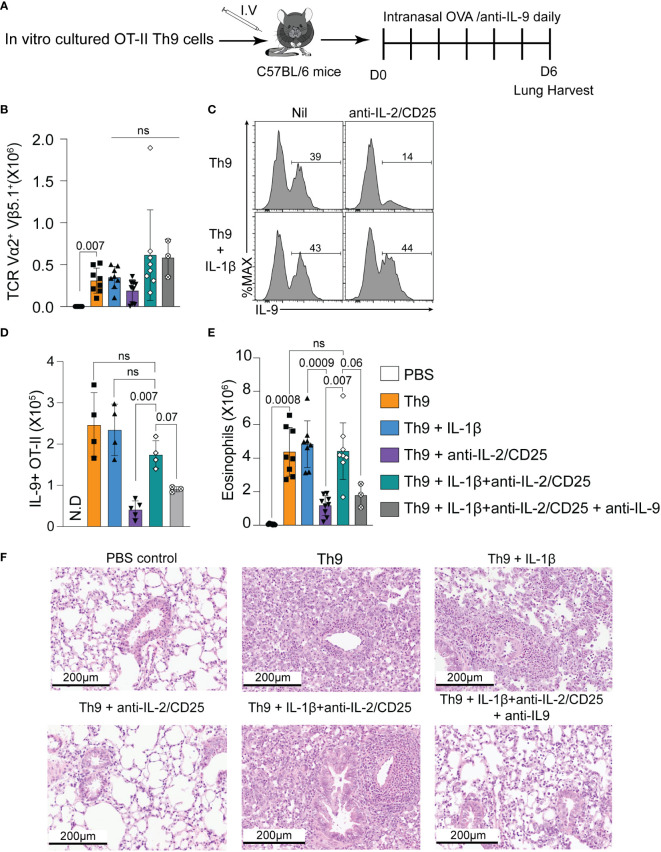
IL-1β rescues the *in vivo* IL-9-dependent inflammatory capacity of IL-2-deprived Th9 cells. **(A)** Schematic of the experimental design of the Ovalbumin (OVA)-induced allergy model after adoptive transfer of indicated Th9 cells. **(B)** Total number of Vα2/Vβ5^+^ OT-II CD4 T cells in the lung 24 hours post-final intranasal OVA exposure. **(C)** Representative histograms of IL-9 staining from Vα2/Vβ5^+^ OT-II CD4 T cells isolated from lungs, gating indicates percentage of OT-II cells expressing IL-9. **(D)** Total cell number of IL-9- producing Vα2/Vβ5^+^ OT-II CD4 T cells in each group. Each data point represents one mouse. n= 4 for Th9, Th9+ Il-1β, and Th9+ IL-1β anti-IL-2/CD25. n=5 for Th9+ anti-IL-2/CD25, and n=3 for Th9+ IL-1β anti-IL-2/CD25 + anti-IL-9. N.D: non determined **(E)** Total pulmonary eosinophil numbers in indicated recipient mice. Data are representative of 3-7 mice per group from 2-3 pooled experiments for every group except the anti-IL-9 treated mice. This group was only included in one experiment where there were three mice. **(F)** Representative H&E images of recipient mice lung tissue harvested on day 6 after OVA treatment and adoptive OT-II cell transfer. Data are considered significant if *p* value ≤0.05 in a one-way ANOVA analysis with a Tukey post-test. Error Bars indicate standard deviation. ns, non-significant.

Given the role of Th9 cells during allergic inflammation (2), we asked whether IL-1β could rescue the inflammatory potential of Th9 cells differentiated under IL-2-limiting conditions. To this end, we employed an ovalbumin (OVA)-induced lung allergy model in which the adoptive transfer of ovalbumin-specific CD4 T cells (OT-II cells) and intranasal treatment with ovalbumin and TSLP results in the induction of lung allergy in a Th9-dependent manner as previously shown (13). In our experiments, OT-II cells were cultured under various Th9-polarizing conditions (Th9, Th9+IL-1β, Th9 anti-IL-2/CD25, Th9 anti-IL-2/CD25+IL-1β) and transferred intravenously (i.v.) prior to intranasal OVA exposure (**Figure 3A**). An additional group of mice received anti-IL-9 to determine the requirement of IL-9 for pulmonary inflammation. After OVA treatment, all OT-II Th9 recipient mice had similar numbers of Vα2/Vβ5.1^+^ OT-II cells (*p*>0.05 for all groups), indicating that the *in vitro* polarizing conditions had minimal impact on Th cell proliferation *in vivo* after OVA exposure (**Figure 3B**). Similar to our *in vitro* data, IL-2-deprived Th9 cells exhibited reduced capacity to produce IL-9 as compared to standard Th9 cells after *in vivo* intranasal OVA challenge. Again consistent with our previous findings, the frequency and total numbers of pulmonary IL-9-producing OT-II cells were increased in mice that received IL-1β-treated IL-2-deprived Th9 cells as compared to mice that received untreated IL-2-deprived cells (**Figures 3C, D**), indicating that IL-1β rescues the *in vivo* capacity of Th9 cells to maintain IL-9 production when primed in IL-2-limiting conditions. In terms of Th9-induced eosinophilic inflammation, mice receiving Th9 cells cultured under IL-2 limiting conditions had reduced pulmonary eosinophil numbers as compared to IL-2 replete Th9 and Th9+IL-1β controls (**Figure 3E**). In contrast, mice receiving Th9 anti-IL-2/CD25+IL-1β cells exhibited a fully rescued eosinophil response, and this effect was abrogated by *in vivo* neutralization of IL-9 (**Figure 3E**). This is consistent with the lung histology data obtained after hematoxylin and eosin (H&E) staining, which show that Th9 cells induce pulmonary inflammation as compared to PBS controls and this is similar in mice that received Th9 cells cultured with IL-1β (**Figure 3F**). These data likely suggest that our standard Th9 conditions already exhibit maximal NF-kB activity and that their *in vivo* inflammatory capacity is not enhanced by addition of exogenous IL-1β. In contrast, mice that received IL-2-deprived Th9 cells exhibited dramatically reduced airway pathology that was similar to PBS controls. Transfer of IL-2 deprived Th9 cells treated with IL-1β, however, had similar levels of pathology as compared to standard Th9 recipient mice. Importantly, the group of mice that received IL-1β-rescued Th9 cells with *in vivo* anti-IL-9 blockade exhibited reduced levels of inflammation (**Figure 3F**) indicating that both lung eosinophilia and airway inflammation are IL-9-dependent. Together, our data suggest that IL-1β restores the *in vivo* IL-9-dependent inflammatory capacity of IL-2-deprived Th9 cells.

### IL-2/STAT5 represses IL-1R1 expression

**Figure 4:**
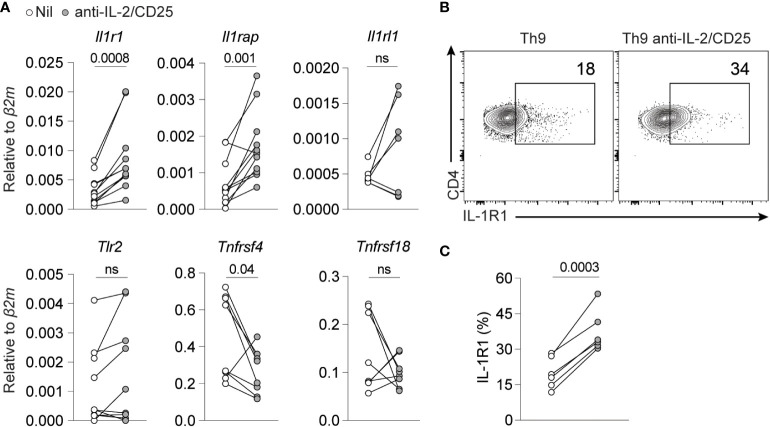
IL-2 limiting conditions enhance IL-1R1 expression on Th9 cells. **(A)** NF-KB-inducing cell surface and cytokine receptor gene expression in standard Th9 or IL-2-deprived Th9 cells. Each point represents data from one mouse. The combined data represent at least a n=7 mice. **(B)** Representative contour plots of cell surface IL-1R1 expression on Th9 and IL-2-deprived Th9 cells on day 5 of culture. **(C)** Quantification of IL-1R expression from panel **(B)** Data are representative of Th9 cultures from 6-7 mice pooled from 3 individual experiments. Data were considered significantly different from paired *t*-test at p value ≤0.05. ns, non-significant.

IL-1β was uniquely able to rescue Th9 differentiation when IL-2 was limited as compared to other NF-kB-activating cytokines or receptors. As many of these receptors share common downstream signaling pathways (i.e., MyD88, TRAF6 and NF-kB), we reasoned that this may be due to differences in cytokine or TNF-family receptor expression under IL-2 limiting conditions. Indeed, we observed that mRNA expression of components of the IL-1β receptor (*Il1r1* and *Il1rap*) was upregulated when IL-2 was limiting whereas the IL-33 receptor (*Il1rl1*), *Tlr2*, and GITR (*Tnfrsf18*) were unchanged in these conditions (**Figure 4A**). Interestingly, OX40 (*Tnfrsf4*) was significantly reduced, which potentially explains why it was not consistently able to rescue Th9 differentiation under these conditions (**Figure 2**). Importantly, we also observed enhanced cell surface protein expression of IL-1R1 when IL-2 was limiting (**Figures 4B, C**). These data together support a model where cells that differentiate in IL-2-limiting conditions upregulate IL-1R1 that, in turn, promotes Th9 differentiation *via* NF-kB.

### IL-1β enhances Th9 IL-2 responsiveness

**Figure 5:**
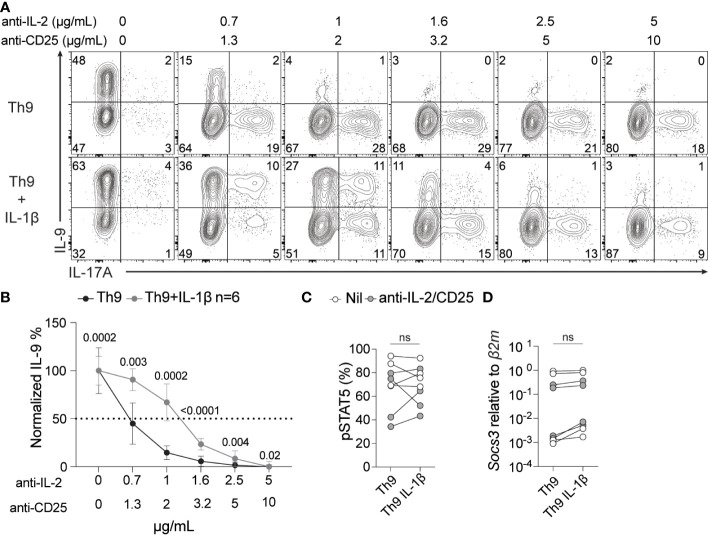
IL-1β signaling enhances IL-2 sensitivity during Th9 differentiation. Th9 cells were cultured in the presence or absence of IL-1β and increasing amounts of anti-IL-2/CD25 and IL-9 and IL-17A were measured by ICS at day 5 of culture. **(A)** Representative contour plots of intracellular IL-9 and IL-17A after restimulation with PMA and ionomycin. **(B)** Quantification of %IL-9^+^ Th cells at day 5 of culture from panel A, p<0.05 by paired Student’s t-test was considered significant, error bars represent SD. pSTAT5 protein **(C)** and *Socs3* mRNA **(D)** levels of Th9 cells with or without IL-1β at no IL-2/CD25 blockade (Nil) or at 1μg/ml anti-IL-2/2μg/ml anti-CD25 (anti-IL-2/CD25). Data in panel A and B are representative of naïve T cell cultures from 6 mice and pSTAT5 and *Socs3* data are representative of cultures from 4-6 mice from 2 pooled experiments. ns, non-significant.

IL-1 signaling has been previously shown to enhance IL-6/STAT3 signaling in Th17 cells by repressing suppressor of cytokine signaling (SOCS3) (56). SOCS3 has also been recently implicated in repressing IL-2 or IL-7-driven STAT5 signaling in Tregs (57). Based on these previous studies, we hypothesized that IL-1β may enhance Th9 IL-2 responsiveness *via* a similar mechanism. To test this, we initially titrated anti-IL-2 and anti-CD25 antibodies to generate culture conditions with decreasing degrees of IL-2 availability and subsequently measured IL-9 production from these cells. With standard Th9 conditions we observed an expected dose-dependent decrease in IL-9 (**Figures 5A, B**) and a dose-dependent increase in IL-17A with limiting IL-2 availability that was consistent with what we have previously shown (16) (**Figure 5A**). In Th9 cells cultured with IL-1β, we still observed a dose-dependent decrease in IL-9 production, however, these cells were more resistant to limited IL-2 availability (e.g. it required ~2-fold more IL-2/CD25 blocking antibodies to mitigate IL-9 production) than cells cultured without IL-1β (**Figures 5A, B**). We then asked if this enhanced resistance to limited IL-2 availability coincided with reduced SOCS3 expression and/or elevated phospho-STAT5 (pSTAT5). However, we did not observe changes in either *Socs3* mRNA or pSTAT5 in the presence or absence of IL-1β in standard or IL-2-deprived Th9 conditions (**Figures 5C, D**). These data suggest that while IL-1β enhances the sensitivity of Th9 cells to limiting amounts of IL-2, this likely occurs through SOCS3-/pSTAT5-independent mechanisms.

### IL-1β promotes Th9 differentiation in IL-2-limiting conditions by suppressing the expression of BCL6

**Figure 6:**
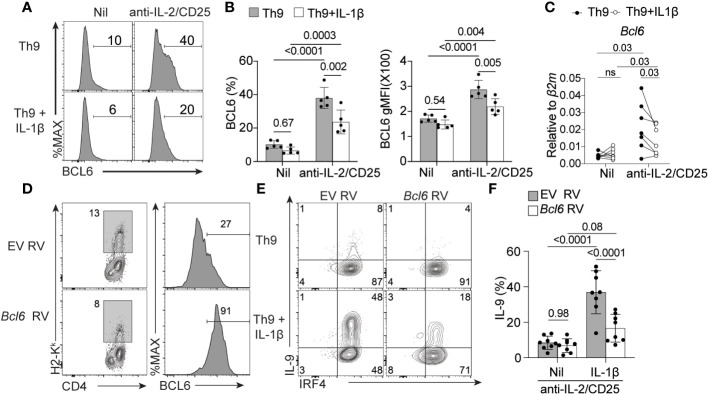
IL-1β reduces the expression of BCL6 to rescue IL-9 production in IL-2-limiitng conditions. **(A)** Representative histograms of BCL6 expression in Th9 cells with and without IL-2 deprivation and with and without IL-1β as determined by flow cytometry. **(B)** Quantification of %BCL6^+^ Th cells (left panel) and relative BCL6 protein expression (geometric mean fluorescence intensity (MFI), right panel).**(C)***Bcl6* mRNA relative expression in Th9 cells cultured as in panel **(A)**. **(D)** Representative histograms depicting empty vector retrovirus (EV RV) or *Bcl6*-expressing retrovirus (*Bcl6* RV) transduction efficiency of Th9 cells cultured under IL-2 limiting conditions and associated BCL6 protein expression from transduced H2-K^k+^ cells). **(E)** Representative contour plots of intracellular IRF4 and IL-9 staining of IL-2-deprived Th9 cells in the presence or absence of IL-1β that have been transduced with empty vector or *Bcl6*-expressing retrovirus. **(F)** Quantification of data in panel **(E)** Data represent naïve T cell cultures from 5-8 mice per group from 2-3 individual experiments. Statistical significance (*p*<0.05) was determined by one-way ANOVA. Error bars represent SD. ns, non-significant.

Previous work has shown that BCL6 expression is repressed by IL-2/STAT5 signaling and BCL6 binding to the *Il9* locus results in reduced *Il9* expression and Th9 cell differentiation (14, 15). In line with these findings, we showed that *Bcl6* mRNA was also elevated in Th9 cells cultured under IL-2-limiting conditions (**Figure 1D**). Here, we questioned whether IL-1β-mediated rescue of IL-9 production correlated with changes in BCL6 expression. As expected, BCL6 protein levels increased in Th9 cells cultured under IL-2 deprivation, validating our mRNA analyses (**Figures 6A-C**). Further, addition of IL-1β to IL-2-deprived Th9 cells resulted in a significant reduction of BCL6 mRNA and protein as compared to IL-2-deprived Th9 cells (**Figures 6A-C**). These data suggest that IL-1β suppresses BCL6 that would normally inhibit IL-9 production when IL-2 is limiting. To test this possibility, we asked if maintaining high levels of BCL6 expression in IL-1β-treated cells would prevent the rescue of Th9 differentiation. To this end, we transduced CD4 T cells with an empty vector retrovirus (EV RV) or BCL6-expressing retrovirus (Bcl6 RV) at 24 hours post-initiating Th9 anti-IL-2/CD25 cultures in the presence or absence of IL-1β. As expected, Th9 cells transduced with BCL6 RV had heightened BCL6 expression as compared to EV controls (**Figure 6D**) and reduced production of IL-9 in IL-2-limiting conditions (**Figures 6E, F**), validating the suppressive role of BCL6 in Th9 cell differentiation (14, 15). Interestingly, while IL-1β enhanced IL-9 production in EV RV-transduced cells, this was dramatically reduced in cells transduced with BCL6 RV (**Figures 6E, F**). Together, these data strongly suggest that IL-1β promotes IL-9 production through its repressive effects on BCL6.

## Discussion

The *in vitro* differentiation of IL-9-producing Th9 cells requires IL-2/STAT5 signaling as IL-2 deficiency or IL-2 blockade results in a complete loss of IL-9 producing cells (15, 16, 43), and the induction of a population of IL-17-producing Th17-like cells (16). Paradoxically, previous work indicates that Th9 cells are often prevalent, or exert their functions, in inflammatory environments with poor IL-2 bioavailability (i.e. tumors, chronically inflamed tissues) and co-develop with IL-2-sequestering Tregs and IL-2-suppressed Th17 cells (17–22). These data indicate that other signals may promote the Th9 phenotype and function in IL-2-limiting environments *in vivo*.

In this work, we identify a novel circuit that acts to enhance or maintain Th9 cell function within IL-2-limited environments driven by the inflammatory cytokine IL-1β. In this circuit, we show that IL-2 deprivation results in a dramatic reduction of the Th9 NF-kB transcriptional signature and a reciprocal increase in the expression of the IL-1 receptor (IL-1R1) that enhances Th9 cell responsiveness to exogenous IL-1β. Reinvigoration of the NF-kB pathway in IL-2-deprived Th9 cells with IL-1β restores their capacity to produce IL-9 by repressing the expression of the Th9 inhibitory transcription factor BCL6. These data suggest that Th9 cells may compensate for limited IL-2/STAT5 signaling in inflamed tissues by enhancing their responsiveness to IL-1β.

Our data indicate that IL-2/STAT5 signaling is critical for inducing the NF-kB pathway during Th9 cell differentiation. However, how IL-2/STAT5 signaling interfaces with NF-kB activation is not well understood. Our current understanding of these pathways in T cells largely comes from studies interrogating NF-kB/IL-2 synergy in T cell activation after T cell receptor (TCR) engagement. Within this setting, TCR/CD28 engagement results in the downstream activation of NF-kB which then directly transactivates the IL-2 gene and induced T cell proliferation and differentiation (27). Examples of how IL-2 may impact NF-kB signaling, however, are more limited. Stimulation of human monocytes with IL-2 resulted in enhanced NF-kB DNA binding capacity (58) and IL-2-induced NK cell cytolytic activity was largely abrogated after treatment of NF-kB inhibitors (59, 60). These studies suggest that IL-2-mediated activation of NF-kB may be common between lymphocyte populations and conserved between mouse and human immune cells. Defining the mechanisms of this interaction will provide further insight into how IL-2 promotes cell proliferation and function, and our work suggests that NF-kB activity could potentially be compensated for by NF-kB-activating cytokines when IL-2 is limiting.

NF-kB-activating cellular receptors and cytokines are often elevated in inflamed tissues and have been previously shown to enhance Th9 cell differentiation and function. For example, the NF-kB-activating receptors OX40 and GITR drive the conversion of Tregs to the Th9 phenotype which is consistent with the role of these receptors in driving allergic disease and anti-tumor immunity (3, 34). Additionally, the NF-kB-inducing cytokines TNF, TL1A and IL-1β all promote Th9 cell differentiation and specifically enhance the anti-tumor efficacy of Th9 adoptive cellular therapy (ACT) (31–33, 61). Despite the fact that NF-kB-activating receptors and cytokines almost universally promote the Th9 phenotype, we found that only IL-1β was able to consistently rescue Th9 cell differentiation under IL-2 limiting conditions *in vitro* and IL-9-mediated allergic inflammation *in vivo*. Mechanistically, we showed that IL-2 limiting conditions enhanced expression of IL-1R1 and the co-receptor IL-1RAP at both the mRNA levels and IL-1R1 at the cell surface protein level, implicating that IL-2 deprivation uniquely enhances the sensitivity of Th9 cells to IL-1β. The differentiation of both T follicular helper cells (TFH) and Th17 cells is suppressed by IL-2/STAT5 signaling and correlates with high levels of IL-1R1 expression (56, 62). IL-1β/IL-1R1-signaling also enhances the capacity of these Th subsets to produce their signature functions or cytokines. For example, T follicular helper cells express minimal IL-2Rα (CD25) and high levels of IL-1R1 expression and treatment with exogenous IL-1β after protein vaccination enhances TFH function in mice (62). For Th17 cells, IL-1R1 is induced through IL-6 signaling (63), and we and others showed that IL-6/STAT3-signaling in Th cells results in diminished IL-2/STAT5 responsiveness (42, 64). Addition of exogenous IL-1β to Th cell cultures enhanced *in vitro* Th17 cell differentiation and IL-1R1-deficient T cells failed to induce Th17-driven disease in murine experimental autoimmune encephalomyelitis (EAE) (63). These findings suggest that IL-2-limiting conditions may universally promote IL-1R1 expression and enhance Th cell functionality to enhance protective immunity or drive disease.

Similar to IL-1β, IL-1α also signals through IL-1R1, induces NF-kB activation *via* MyD88 (65, 66) and enhances IL-9 production in standard Th9 cells (67). These findings suggest that IL-1α may also be able to rescue IL-9 production under IL-2 limiting conditions. However, in a pilot experiment, we found that IL-1α did not consistently rescue IL-9 production in IL-2-deprived Th9 cells (data not shown). These data suggest that IL-1β and IL-1α may have differing capacities to rescue IL-9 based on differences in their intracellular signaling. Previous work has shown that IL-1β and IL-1α interact differently with the IL-1R due to their differential lectin capacity, which may result in differences in cell signaling despite using the same receptor (68). Further, IL-1α and IL-1β exhibit divergent roles in several *in vivo* models of inflammation (66, 69, 70). Thus, it is possible that these related cytokines may also differentially regulate IL-9 production in IL-2 limiting conditions. However, the mechanisms that may regulate these differences are not directly forthcoming. Additional work is required to determine how these different IL-1 family members elicit distinct responses in this setting.

In the anti-tumor setting, culture of Th9 cells with IL-1β or cells with heightened NF-kB activity exhibited enhanced expression of the CD4 cytotoxic lymphocyte transcription factor Eomes and enhanced granzyme/perforin-associated killing after adoptive transfer (31, 61). In addition, IL-1β-treated Th9 cells also induced the expression of the transcription factor IRF1 which enhanced their capacity to produce both IL-9 and IL-21. Interestingly, anti-tumor activity of Th9 cells in these studies was dependent on IL-21, but not IL-9. In this tumor model, the enhanced anti-tumor effects of IL-1β-treated Th9 cells were indirect and required IL-21 priming of CD8 T cells and natural killer (NK) cells (51). In contrast, we found that IL-1β treatment rescued the *in vivo* inflammatory potential of Th9 cells in an allergic airway model of disease in an IL-9-dependent manner. These differences in IL-9 vs. IL-21-dependency likely stem from the model system used (i.e. allergic airway disease in our study vs. cancer), especially as CD8 T cells and NK cells are minimally involved in the Th9-mediated allergic response. Indeed, Th cell-derived IL-9 is critical for the induction of the allergic response through the recruitment of mast cells and eosinophils to the lung *via* an airway macrophage intermediate (71). These data suggest that IL-1β may differentially modulate Th9 activity during allergy (via maintaining IL-9 production) and cancer (by induction of IL-21).

Mechanistically, we show that IL-1β rescues the production of IL-9 by IL-2-deprived Th9 cells by repressing the expression of the Th9 inhibitory transcription factor BCL6. As BCL6 competes for STAT5 binding at the *Il9* locus, our data suggest that IL-1β-mediated repression of BCL6 enhances the capacity of STAT5 to bind the locus when IL-2/STAT5 signaling is limited. However, how IL-1β mediates repression of BCL6 in these settings remains unclear. In macrophages, BCL6 is a suppressor of inflammatory activity where BCL6-deficient macrophages exhibit heightened and dysregulated cytokine production after stimulation *via* NF-kB activating receptors (i.e. LPS/TLR4) (72). Macrophage NF-kB and BCL6-induced regulomes intersected and exhibited potential antagonism where NF-kB activation limited the BCL6 regulome. Intriguingly, this may also be through direct suppression of BCL6 as NF-kB p65 directly bound several conserved non-coding regions surrounding the *Bcl6* gene (72). While IL-1β-induced NF-kB suppresses BCL6 expression, BCL6 also appears to reciprocally suppress NF-kB activation. Macrophage BCL6 and its co-repressor binding is enriched surrounding NF-kB-regulated genes and disruption of BCL6/co-repressor interactions results in elevated inflammatory response gene expression (72, 73). Based on these data, it is intriguing to speculate that the reduced NF-kB signature that we observed in IL-2-deprived Th9 cells may be regulated by BCL6. These data suggest that BCL6 inhibitors may be beneficial in enhancing NF-kB signaling and Th9 function, especially in response to chronic antigens and in tumor draining lymph nodes tumors where Th cell BCL6 expression is elevated (74, 75).

Together, our data define an IL-1β-NF-kB-BCL6 axis that governs Th9 differentiation in IL-2-limiting conditions. As Th9 cells play important roles in auto- and anti-tumor immunity, our studies suggest that therapeutic targeting IL-1β/IL-1R1 signaling may be beneficial in dampening Th9-mediated inflammation or may be bolstered to enhance tumor clearance.

## Data availability statement

The datasets presented in this study can be found in online repositories. The names of the repository/repositories and accession number(s) can be found below: https://www.ncbi.nlm.nih.gov/geo/, GSE41317.

## Ethics statement

The animal study was reviewed and approved by Purdue Animal Care and Use Committee.

## Author contributions

DC and MC performed the *in vitro* experiments. DC performed *in vivo* experiments. BY completed the bioinformatic analyses. DC, CC and ZI performed q-RT-PCR. NA and SP assisted with tissue preparation. DC analyzed the results. AD provided reagents and insight in BCL6 studies. MK and MO contributed to the writing of the paper and conceived the study. All authors contributed to the article and approved the submitted version.

## Funding

This work was supported by startup funds (Purdue University) and a Showalter Trust Award (41000747) to MO, and an NIGMS R35GM138283 to MK. DC was supported by a Purdue University Ross-Lynn Graduate Student Fellowship and a Purdue College of Science Bilsland Fellowship. BY was supported by the SIRG Graduate Research Assistantships Award from the Purdue University Center for Cancer Research, P30CA023168 and a Purdue College of Agriculture Bilsland Fellowship.

## Acknowledgments

The authors would like to acknowledge Dr. Mark H. Kaplan for critical review of the manuscript and Ms. Tripti Bera for her technical assistance in these studies.

## Conflict of interest

The authors declare that the research was conducted in the absence of any commercial or financial relationships that could be construed as a potential conflict of interest.

## Publisher’s note

All claims expressed in this article are solely those of the authors and do not necessarily represent those of their affiliated organizations, or those of the publisher, the editors and the reviewers. Any product that may be evaluated in this article, or claim that may be made by its manufacturer, is not guaranteed or endorsed by the publisher.
